# Patterns of preference importance ratings among African-American and White nursing home residents

**DOI:** 10.1093/geroni/igaa057.3063

**Published:** 2020-12-16

**Authors:** Nytasia Hicks, Katherine Abbott, Allison Heid, Kendall Leser, Kimberly Van Haitsma

**Affiliations:** 1 Miami University, Oxford, Ohio, United States; 2 Ardmore, Pennsylvania, United States; 3 Penn State University, University Park, Pennsylvania, United States

## Abstract

Background: The Preferences for Everyday Living Inventory (PELI) was developed to assess the psychosocial preferences of older adults receiving home care (PELI-HC) and then revised for nursing home residents (PELI-NH). While the PELI-HC has been tested to identify patterns in preference ratings by race, the PELI-NH has not. We sought to explore whether the PELI-NH tool captures differences in preference ratings of African-American and White NH residents. Methods: Preference assessment interviews were conducted with NH residents (n = 317). Analysis via a Mann-Whitney U test, results show that 46 of 72 (63.88%) a preference importance items were not statistically different between African-American and White NH residents. Additionally, African-Americans reported greater importance than White older adult NH residents in 26 of 72 (36%) preference importance items. Conclusion/Implications: It appears that the PELI-NH can test group differences in preference importance among African-American and White NH residents; implications for practice will be discussed. Part of a symposium sponsored by the Research in Quality of Care Interest Group.

